# Growth mindset and learning engagement among primary school students: a moderated sequential mediation model via grit and effort belief under perceived teacher support

**DOI:** 10.3389/fpsyg.2026.1891828

**Published:** 2026-09-04

**Authors:** Wei Liang, Huarong He, Lingyi Peng, Youzhi Liang, Song Tong, Pan Lin

**Affiliations:** 1 Department of Psychology, School of Educational Science, Hunan Normal University, Changsha, China; 2 Hunan First Normal University, Changsha, China; 3 School of Humanities and Management, Ningxia Medical University, Yinchuan, China; 4 China Central Academy of Fine Arts, Beijing, China; 5 Bay Area School of Applied Psychological Sciences, Beijing Normal University, Zhuhai, China; 6 Beijing Key Laboratory of Applied Experimental Psychology, National Demonstration Center for Experimental Psychology Education, Faculty of Psychology, Beijing Normal University, Beijing, China

**Keywords:** effort beliefs, growth mindset, learning engagement, motivation, primary school students, teacher support

## Abstract

**Background:**

Growth mindset is often theorized to energize learning engagement; however, belief rarely translates directly into sustained participation. We, therefore, examined whether a growth mindset fosters engagement by first strengthening perseverance toward long-term goals (grit) and, in turn, consolidating the belief that effort effectively improves ability (effort belief), and whether this motivational pathway depends on perceived teacher support.

**Methods:**

Using cluster sampling, 1,959 primary school students completed measures of growth mindset, learning engagement, grit, effort belief, and perceived teacher support.

**Results:**

Growth mindset was positively associated with learning engagement. Both grit and effort belief mediated the association, and the indirect effect through grit, followed by effort belief, was significant. Perceived teacher support moderated the path from growth mindset to grit and the direct path from growth mindset to learning engagement.

**Discussion:**

The findings support a sequential motivational conversion process: growth mindset→grit→effort belief→learning engagement, and identify perceived teacher support as a boundary condition that strengthens the translation of growth-oriented beliefs into persistent, engaged learning behavior.

## 1 Introduction

Learning engagement is not merely time on task; it reflects a coordinated system of sustained behavioral participation, emotional investment, and cognitive focus that supports achievement and developmental trajectories (Fredricks et al., 2004; Schaufeli et al., 2002). In elementary school, engagement is especially consequential because it scaffolds knowledge construction, consolidates learning habits, and lays the groundwork for lifelong learning. However, engagement remains vulnerable to fluctuations in motivation and avoidance tendencies triggered by academic setbacks, underscoring the need to identify psychological mechanisms that help sustain engagement during this formative period (Fredricks et al., 2016). A central question is therefore not only which students engage, but how engagement is cultivated and maintained in the face of difficulty.

Growth mindset provides one candidate lens for this conversion problem. Mindset theory proposes that beliefs about the malleability of intelligence shape how students interpret challenge, effort, and failure, thereby influencing persistence and learning behaviors (Dweck, 2006; Dweck and Leggett, 1988). Individuals endorsing a growth mindset tend to exhibit greater challenge-seeking and persistence, particularly among adolescents (Blackwell et al., 2007; Yeager et al., 2019). However, meta-analytic evidence reveals heterogeneous and often modest direct effects of mindset interventions (Sisk et al., 2018; Macnamara and Burgoyne, 2022), suggesting that mindset does not unilaterally “cause” academic outcomes. Instead, its influence likely operates through intermediate motivational processes and is contingent upon contextual supports—an issue particularly salient among primary school students, whose motivational systems remain highly malleable yet understudied.

Although closely related, growth mindset and effort beliefs are conceptually distinct and operate at different levels of the motivational architecture. Growth mindset represents a global implicit theory concerning the malleability of human attributes (Dweck, 2006); it is a top-down belief system about what is possible. Effort beliefs, by contrast, constitute a proximal cognitive appraisal regarding whether expending effort is effective, necessary, and worthwhile in a particular learning context (Blackwell et al., 2007; Eccles and Wigfield, 2002). In other words, growth mindset answers the question “Can I improve?” whereas effort beliefs answer “Does it pay to try here and now?” This distinction is critical: holding a growth mindset does not automatically generate stable effort beliefs. Students may intellectually endorse the idea that ability is malleable yet remain unconvinced that their own effort will be productive in the face of repeated failure.

From a social cognitive perspective, growth mindset is likely to influence engagement by shaping the motivational infrastructure that supports sustained action (Bandura, 1997; Ryan and Deci, 2000). One such component is grit—perseverance and sustained passion for long-term goals (Duckworth et al., 2007). Grit functions as the volitional bridge that translates a global mindset into situated effort beliefs. By sustaining action across setbacks, grit allows students to accumulate mastery experiences that validate the utility of effort, thereby transforming an abstract philosophy into a concrete, context-specific belief. When students believe abilities can be developed, setbacks may be reinterpreted as temporary and surmountable, rendering perseverance both rational and worthwhile (Dweck, 2006). Consistent with logic, recent evidence indicates that grit (particularly perseverance of effort) can function as a mediator linking growth mindset to engagement (Zhao and Chang, 2024).

Nevertheless, grit primarily captures the volitional-behavioral dimension of motivation. To understand how students cognitively appraise the value of their actions, researchers have emphasized the effort belief—the conviction that effort is an effective means of improving ability and achieving success (Blackwell et al., 2007). Effort belief represents a proximal cognitive filter through which distal traits are translated into learning behaviors. A critical theoretical issue concerns the causal ordering of grit and effort beliefs. Drawing on trait-based perspectives on grit (Duckworth et al., 2007; Muenks et al., 2017) and feedback models of self-regulation (Carver and Scheier, 2016), we conceptualize grit as a relatively enduring volitional disposition that supports sustained goal-directed effort over time and in the face of challenges. Effort beliefs, by contrast, are construed as comparatively malleable and context-sensitive cognitive appraisals concerning the meaning and effectiveness of effort (Blackwell et al., 2007; Tempelaar et al., 2015). Through repeated cycles of goal pursuit and self-regulatory feedback, students observe the consequences of their persistent efforts and progressively update their beliefs about whether effort is worthwhile and effective. Thus, whereas grit provides a relatively stable dispositional basis for sustained action, effort beliefs may emerge and consolidate through students’ accumulated interpretations of effort-related experiences. In other words, the capacity to grit must be established before stable beliefs about the utility of effort can crystallize. Without this volitional foundation, effort-related cognition remains unstable and less predictive of engagement. This logic justifies modeling grit as an antecedent—rather than a consequence—of the belief in effort in the motivational chain linking mindset to engagement.

Beliefs and dispositions, however, do not translate into behavior in a psychological vacuum. A situated perspective on mindset emphasizes that the classroom norms and interpersonal signals can either activate or suppress the functional consequences of growth beliefs (Yeager et al., 2019). Perceived teacher support, encompassing emotional care, academic assistance, and autonomy support (Jia et al., 2009), is consistently linked to engagement and functions as a key “conversion context” (Tao et al., 2022; Vargas-Madriz et al., 2024). In supportive environments, when mistakes are treated as part of learning, students with a growth mindset may feel safer taking risks, persist longer, and have their effort beliefs validated through feedback (Yeager et al., 2022). By contrast, in low-support contexts, fear of evaluation and threat cues may dampen persistence and narrow the motivational bandwidth through which mindset can influence engagement. These considerations motivate testing perceived teacher support as a moderator.

**Figure 1:**
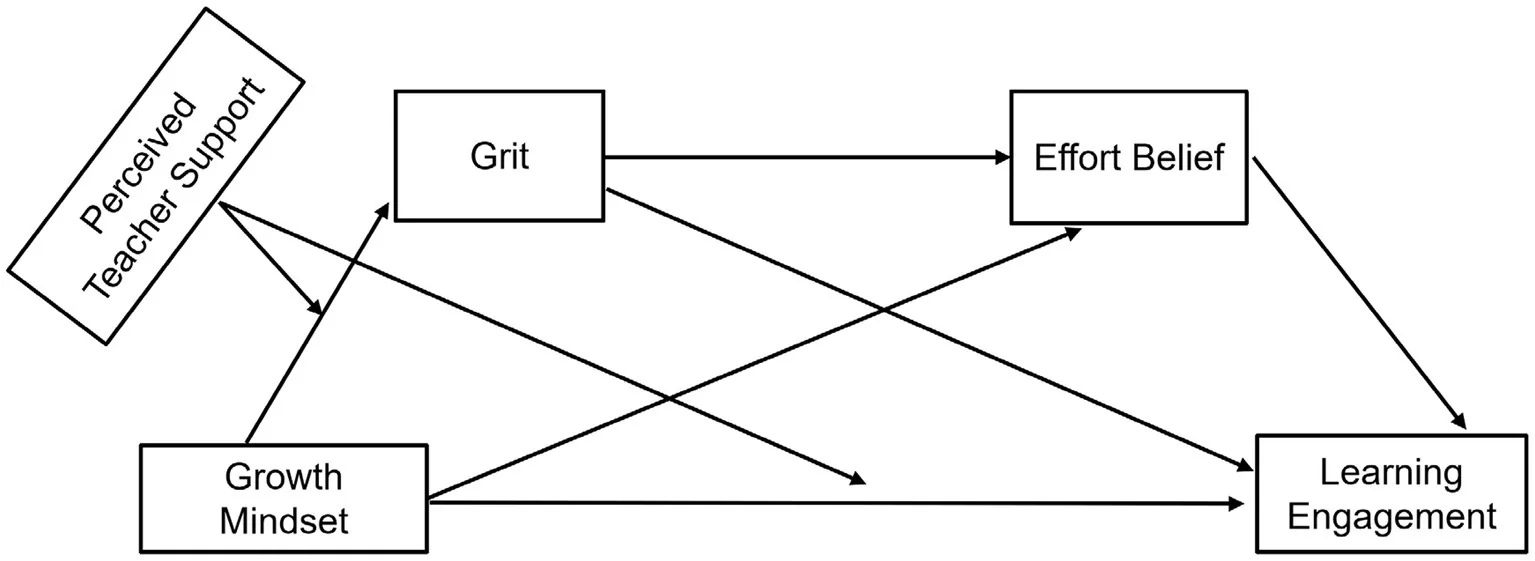
Hypothetical model of moderated chain mediation.

Integrating these perspectives, the present study advances a moderated sequential mediation model (Figure 1). We propose that growth mindset predicts learning engagement through the serial mediators of grit and effort beliefs, with perceived teacher support moderating both the mindset–grit pathway and the direct association between mindset and engagement. Based on this framework, we hypothesize:

*H1*: Growth mindset positively predicts primary school students' learning engagement.

*H2*: Grit mediates the relationship between growth mindset and primary school students’ engagement.

*H3*: Effort Belief mediates the relationship between growth mindset and primary school students’ engagement.

*H4*: Growth mindset predicts learning engagement through sequential mediation via grit and effort belief.

*H5*: Perceived teacher support moderates the effect of growth mindset on primary school students' grit.

*H6*: Perceived teacher support moderates the effect of growth mindset on primary school students' learning engagement.

By articulating the sequential logic from trait-level perseverance to cognition-level effort appraisal—and embedding this process within supportive classroom contexts—this study aims to clarify how and when growth mindset shapes learning engagement during primary school.

## 2 Methods

### 2.1 Participants

The participants were selected from students in grades 4 to 6 across seven primary schools in H Province. This survey was approved by the Ethics Committee of the author’s university. A cluster sampling method was used, administering the questionnaires in a group setting by classroom (Peng et al., 2025). A total of 2,015 questionnaires were distributed. After excluding invalid responses (e.g., random answering patterns or questionnaires with more than 10% missing data), 1,959 valid questionnaires were retained, yielding a response rate of 97.22%. The final sample consisted of 984 boys (50.23%), 962 girls (49.11%), and 13 participants who left the gender field blank. By grade level, there were 699 fourth graders (35.92%), 626 fifth graders (31.96%), and 634 sixth graders (32.36%). Geographically, 756 students (38.59%) were from urban areas, while 1,194 students (60.95%) were from rural areas.

### 2.2 Measures

#### 2.2.1 Learning engagement scale

The Chinese version of the Utrecht Work Engagement Scale for Students (UWES-S), compiled by Schaufeli et al. (2002) and revised by Fang et al. (2008), was used. This scale has shown good reliability across all items (Fang et al., 2008; Zhang et al., 2020). It consists of 17 items across three dimensions: vigor (e.g., “When I’m studying, I feel full of energy”), dedication (e.g., “I find my studies full of meaning and purpose”), and absorption (e.g., “When I am studying, I am so focused that I forget everything else around me”). A 7-point Likert scale was used, with “1” representing “never” and “7” representing “always.” Exploratory factor analysis supported a unidimensional structure (KMO = 0.977, 60.609% variance, all loadings > 0.653); this is consistent with prior research treating engagement as a holistic indicator (Schaufeli et al., 2002), and the scale showed high internal consistency (Cronbach’s α = 0.959).

#### 2.2.2 Growth Mindset scale

The Mindset Scale developed by Dweck (1999) was used to measure participants’ intelligence mindset. This scale consists of eight items, which can be divided into two subscales: four items measuring a growth mindset (e.g., “People can always substantially change their intelligence level”) and four items measuring a fixed mindset (e.g., “Everyone has a certain amount of ability, and they cannot really do much to change it”). Participants responded using a 6-point Likert scale ranging from 1 (strongly disagree) to 6 (strongly agree). Scores for the growth and fixed mindsets were calculated by averaging responses to the four items in each subscale, with higher scores indicating a stronger growth or fixed mindset. This scale has been widely used in previous research on mindset measurement (Blackwell et al., 2007; Yeager et al., 2019) and has been validated by Chinese scholars as having good reliability and validity (Zhao et al., 2022; Cai et al., 2024). EFA with varimax rotation yielded two distinct factors (KMO = 0.808, 69.844% cumulative variance; growth mindset loadings 0.793–0.861, fixed mindset loadings 0.761–0.868), and the scale demonstrated adequate reliability in the current sample (Cronbach’s α = 0.768).

#### 2.2.3 Grit scale

The Short Grit Scale (Grit-S), developed by Duckworth and Quinn, consists of eight items measuring two dimensions: Perseverance of Effort and Consistency of Interest. In line with the theoretical focus of the present study on students’ sustained effort and persistence in learning, only the four-item Perseverance of Effort subscale was used. This approach is consistent with prior empirical research examining perseverance of effort as a distinct dimension of grit in the Chinese context (Zeng et al., 2022). The scale uses a 5-point Likert scale, where 1 represents “not at all like me,” and 5 represents “very much like me.” Higher total scores indicate greater grit. Previous studies have demonstrated the applicability of this measure among Chinese adolescent populations (Wang, 2016; Li et al., 2023). EFA confirmed a single-factor structure (KMO = 0.773, 57.891% variance, all loadings > 0.734), with adequate internal consistency (Cronbach’s α = 0.785).

#### 2.2.4 Effort belief scale

The effort belief scale was adapted from Blackwell et al. (2007). However, only the negatively worded items (five items; e.g., “If I’m not good at something, working hard will not make me better at it”) were used in this study. These items were reverse-scored, so that higher scores indicate stronger effort beliefs. This scale has demonstrated good reliability in previous research (Schürer et al., 2025). EFA indicated a unidimensional structure (KMO = 0.824, 55.696% variance, loadings 0.472–0.827), and the scale showed good internal consistency (Cronbach’s α = 0.908 after reverse-scoring).

#### 2.2.5 Perceived teacher support scale

The Perceived Teacher Support subscale from the School Climate Scale for Adolescents developed by Jia et al. (2009) was used (e.g., “My teachers care about me”). It consists of 7 items, and a 4-point rating scale was used, where 1 represents “never” and 4 represents “always.” The average score of all items was calculated, with higher scores indicating that adolescents perceived greater teacher support. This scale has been widely used in previous research and has shown high reliability and validity (Hou et al., 2018; Chen and Wu, 2024). EFA supported a single-factor solution (KMO = 0.911, 65.648% variance, all loadings > 0.581), with acceptable internal consistency (Cronbach’s α = 0.757).

### 2.3 Data processing and analysis

All analyses were conducted using SPSS 26.0. All variables in this study were self-reported. First, Harman’s single-factor test was applied to assess common method bias. Second, we computed and transformed the key variables before conducting preliminary analyses. Finally, we performed two mediation analyses using the PROCESS macro (version 4.2) for SPSS, specifically Models 6 and 86 (Hayes and Bolin, 2022). After excluding cases with missing values in any analysis variable, the final analytic sample comprised 1,946 participants.

In both models, growth mindset served as the independent variable (X), learning engagement as the outcome (Y), and grit (M1) and effort belief (M2) as mediators. Covariates included gender, grade, and school area. The Bootstrap method (with 5,000 resamples) was used to estimate the bias-corrected confidence intervals for all regression coefficients, providing a robust assessment of the stability and precision of the observed associations within the specified model.

## 3 Result

### 3.1 Preliminary data analysis

To evaluate potential common method bias, we performed Harman’s single-factor test. The first unrotated factor accounted for 31.09% of the total variance, well below the critical threshold of 40%, indicating that common method bias was unlikely to have substantially influenced the results (Podsakoff et al., 2003).

As shown in Table 1, bivariate associations were observed among the study variables. Learning engagement was significantly and positively correlated with growth mindset (*r* = 0.423, *p* < 0.001), grit (*r* = 0.279, *p* < 0.001), effort belief (*r* = 0.336, *p* < 0.001), and perceived teacher support (*r* = 0.438, *p* < 0.001). These correlations indicate patterns of covariation among growth mindset, grit, effort belief, perceived teacher support, and learning engagement in this elementary school student sample.

**Table 1:** Means, standard deviations, and Pearson’s *R* correlations for all variables (*n* = 1959).

Variable	M (total score)	*SD*	1	2	3	4	5	6	7	8
1. School area	1.61	0.488	—							
2. Gender	0.490	0.500	0.050*	—						
3. Grade	4.970	2.420	0.093**	0.018	—					
4. Growth mindset	34.280	7.505	−0.123**	0.005	0.118**	—				
5. Grit	14.540	3.615	−0.136**	0.001	0.008	0.315**	—			
6. Effort belief	25.316	1.270	−0.115**	0.010	0.043	0.451**	0.463**	—		
7. Perceived teacher support	20.580	5.718	−0.150**	−0.084**	0.039	0.364**	0.199**	0.239**	—	
8. Learning engagement	73.540	22.803	−0.168**	−0.060**	0.066**	0.423**	0.279**	0.336**	0.438**	—

Gender was treated as a ‌categorical variable‌ in the analysis, coded as 1 (male) and 0 (female). Reported means are the averages of scale total scores (sum of items), not item-level means.*p < 0.05 and **p < 0.01.

### 3.2 The mediating effect test

Prior to mediation analysis, we standardized all variables. Using growth mindset as the independent variable, we tested its indirect effects on learning engagement via grit and effort beliefs using PROCESS v4.2, Model 6 (serial mediation model) with 5,000 bootstrap samples (*N* = 1,946).

As shown in Table 2, growth mindset positively predicted learning engagement [total effect: *β* = 0.421, *p* < 0.001, 95% *CI* (0.381, 0.462)], supporting Hypothesis 1. The direct effect of growth mindset on learning engagement remained significant after accounting for the mediators [*β* = 0.314, *p* < 0.001, 95% *CI* (0.269, 0.358)], indicating partial mediation.

**Table 2:** Chain mediating effect test (*N* = 1946).

Predictive variable	Outcome variable: grit				Outcome variable: effort belief				Outcome variable: learning engagement			
	*β*	SE	*p*	95%CI	*β*	SE	*p*	95%CI	*β*	SE	*p*	95%CI
School area	−0.097	0.022	< 0.001	[−0.139, −0.054]	−0.026	0.019	0.175	[−0.063, 0.012]	−0.099	0.019	< 0.001	[−0.139, −0.059]
Grade	−0.018	0.022	0.405	[−0.061,0.025]	0.002	0.019	0.933	[−0.036, 0.039]	0.032	0.020	0.118	[−0.008, 0.071]
Gender	0.005	0.022	0.985	[−0.037,0.047]	0.009	0.019	0.618	[−0.028, 0.046]	−0.059	0.020	0.003	[−0.098, −0.020]
Growth Mindset	0.305	0.022	< 0.001	[0.276, 0.341]	0.335	0.020	< 0.001	[0.296, 0.374]	0.314	0.023	< 0.001	[0.269, 0.358]
Grit					0.356	0.020	< 0.001	[0.317, 0.394]	0.104	0.023	< 0.001	[0.059, 0.148]
Effort Belief									0.136	0.024	< 0.001	[0.089, 0.184]
*R^2^*	0.109^***^				0.319^***^				0.221^***^			
*F*	59.346^***^				181.881^***^				96.548^***^			

***p < 0.001.

Within this serial mediation framework, three specific indirect effects were examined using bias-corrected bootstrap 95% confidence intervals based on 5,000 resamples:

Ind1 (via grit alone): Growth mindset→Grit →Learning engagement. Growth mindset significantly predicted grit (*β* = 0.305, *p* < 0.001), which in turn significantly predicted learning engagement (*β* = 0.104, *p* < 0.001). The indirect effect was significant (Ind1 = 0.036, 95% BootCI [0.019, 0.054]), supporting Hypothesis 2.

Ind2 (via effort belief alone): Growth mindset→Effort belief→Learning engagement. Growth mindset significantly predicted effort belief (*β* = 0.337, *p* < 0.001), which in turn significantly predicted learning engagement (*β* = 0.136, *p* < 0.001). The indirect effect was significant (Ind2 = 0.047, 95% BootCI [0.026, 0.068]), supporting Hypothesis 3.

Ind3 (serial: grit→effort belief): Growth mindset→Grit→Effort belief→Learning engagement. This serial pathway was also significant [Ind3 = 0.016, 95% BootCI (0.009, 0.023)], supporting Hypothesis 4.

None of the three bootstrap confidence intervals contained zero, confirming that all indirect effects were statistically significant. The full model explained 22.1% of the variance in learning engagement (*R*^2^ = 0.221), indicating that growth mindset facilitates learning engagement through three interrelated pathways: sustained behavioral regulation (grit), adaptive effort belief, and their sequential combination.

### 3.3 Moderated serial mediating effect test

**Figure 2:**
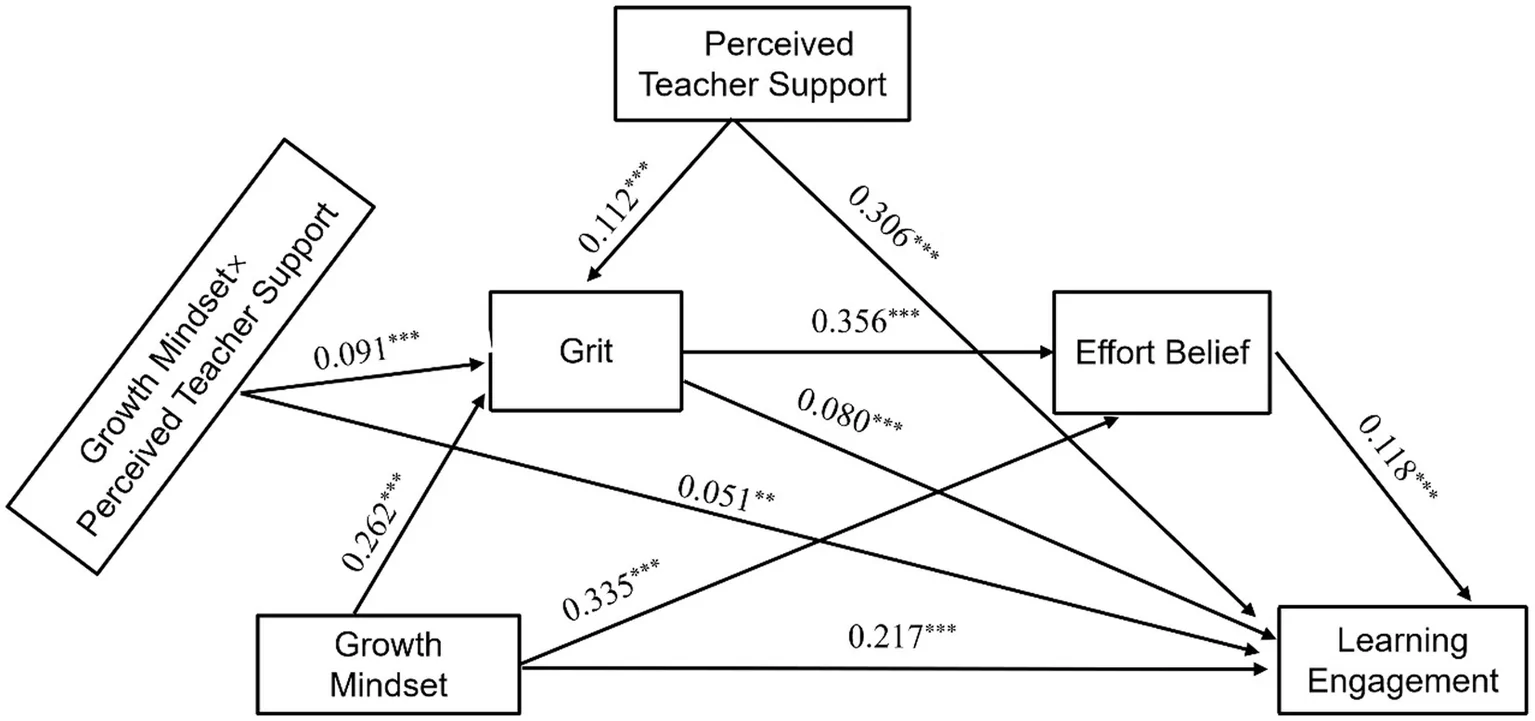
Model of moderated mediation tested using PROCESS 4.2 Model 86. *p* < 0.05 ** *p* < 0.01 *** *p* < 0.001.

Based on the moderated serial mediation analysis (by PROCESS v4.2 Model 86), perceived teacher support significantly moderated the first stage of the mediation pathway from growth mindset to grit (seen in Figure 2); Hypotheses 5 and 6 were supported. Specifically, the interaction term between growth mindset and perceived teacher support significantly predicted grit (seen in Table 3), with a moderation effect coefficient *b* = 0.091, *p* < 0.001, 95% *CI* [0.049, 0.133].

**Table 3:** Moderated chain mediating effect test.

Predictive variable	Outcome variable: Grit				Outcome variable: effort belief				Outcome variable: learning engagement			
	*β*	SE	*p*	95%CI	*β*	SE	*p*	95%CI	*β*	SE	*p*	95%CI
School area	−0.083	0.022	0.001	[−0.126,−0.041]	−0.026	0.019	0.175	[−0.063,0.012]	−0.070	0.019	0.003	[−0.108,−0.032]
Gender	0.008	0.021	0.698	[−0.034,0.050]	0.009	0.019	0.618	[−0.028,0.046]	−0.037	0.019	0.053	[−0.074, 0.001]
Grade	−0.025	0.022	0.246	[−0.068,0.017]	0.002	0.019	0.933	[−0.036,0.039]	0.026	0.019	0.183	[−0.012, 0.063]
Growth mindset	0.262	0.023	<0.0001	[0.216, 0.308]	0.335	0.020	<0.0001	[0.296, 0.374]	0.217	0.023	<0.0001	[0.173, 0.261]
Perceived teacher support	0.101	0.023	<0.0001	[0.055, 0.147]					0.306	0.021	<0.0001	[0.265, 0.347]
Growth Mindset*perceived teacher support	0.091	0.022	<0.0001	[0.049, 0.133]					0.051	0.019	0.008	[0.013, 0.088]
Grit					0.356	0.020	<0.0001	[0.317, 0.394]	0.080	0.022	<0.001	[0.038, 0.123]
Effort belief									0.118	0.023	<0.0001	[0.073, 0.163]
*R^2^*			0.124^***^				0.319^***^				0.307^***^	
*F*			45.581^***^				181.881^***^				107.378^***^	

***p < 0.001.

Simple slope analysis further revealed that the positive effect of growth mindset on grit strengthened as perceived teacher support increased: at a low level of teacher support (−0.998), the effect was *b* = 0.1713, *p* < 0.001; at the mean level (0.002), the effect increased to *b* = 0.262, *p* < 0.001; and at a high level (1.0026), the effect rose further to *b* = 0.3530, *p* < 0.001. Johnson–Neyman analysis indicated that the relationship between growth mindset and grit became statistically significant (*p* < 0.05) when teacher support exceeded approximately −1.841, covering about 93.832% of the sample.

This pattern suggests that perceived teacher support amplifies the indirect effect of growth mindset on learning engagement through grit. In contexts of higher perceived teacher support, growth mindset exerts a stronger influence on grit, thereby enhancing the corresponding indirect pathway (Ind1). However, no significant moderation was found in the latter stage of the serial mediation model—specifically, the interaction between growth mindset/grit and effort belief did not reach significance (*p* = 0.169). Thus, perceived teacher support primarily strenghtens the first-stage link (growth mindset→ grit) rather than uniformly moderating the entire chain.

## 4 Discussion

This study empirically examined the complex relationship between growth mindset and learning engagement among primary school students, incorporating grit and effort belief as serial mediators and considering the moderating role of perceived teacher support. The findings illuminate the motivational pathways through which a growth mindset translates into engaged learning behavior within a supportive classroom ecology.

### 4.1 The impact of growth mindset on learning engagement

Consistent with Hypothesis 1, a significant positive correlation and predictive relationship were found between growth mindset and learning engagement. This aligns with and extends prior research. For instance, longitudinal studies by Kim and Park (2021) demonstrated that lower growth mindset in fourth graders predicted less behavioral engagement over 6 years. Similarly, cross-school studies have confirmed a negative association between a fixed mindset and engagement (Lam et al., 2008; Deci and Ryan, 2013). Theoretically, this link can be explained through two pathways: “challenge transformation” and “psychological empowerment.” Students with a growth mindset reframe academic challenges as “opportunities for cognitive upgrading” rather than “evidence of inability.” This cognitive shift fosters the selection of optimally challenging tasks and the application of metacognitive strategies, such as monitoring “thinking bottlenecks.” Furthermore, by satisfying the core psychological need for autonomy—as outlined in Self-Determination Theory—a growth mindset enhances intrinsic motivation. In autonomy-supportive classrooms where teachers offer a “strategy menu,” students experience heightened choice and competence, facilitating deeper “flow-like” immersion in learning activities. This deep engagement, in turn, reinforces their sense of efficacy, creating a virtuous cycle of “mindset-motivation-behavior.” Consequently, students endorsing a growth mindset exhibit greater persistence, focus, and investment in learning, even when facing potential failure.

### 4.2 The mediating role of grit

The results supported Hypothesis 2, revealing that grit partially mediated the relationship between growth mindset and learning engagement. This finding aligns with Duckworth et al. (2007) conceptualization of grit as the perseverance and passion required for long-term goal attainment. Importantly, within the context of our serial mediation model, grit appears to function as a volitional bridge linking a global cognitive disposition to a specific situational appraisal (effort belief, discussed in Section 4.3). While a growth mindset provides the philosophical premise that abilities can be developed, this “theory” remains inert without the behavioral capacity to persist through challenges.

Our results suggest that students with a growth mindset are more likely to cultivate grit—that is, the tendency to sustain effort and interest over prolonged periods despite adversity. In essence, mindset answers the question “Can I improve?” While grit answers “Am I willing to struggle to improve?” This distinction is crucial for primary school students, who often encounter frustration when transitioning from basic skill acquisition to more complex problem-solving.

This finding is consistent with existing theoretical frameworks. According to implicit theory, individuals with a growth mindset adopt mastery goals (Blackwell et al., 2007), which manifest as sustained passion and perseverance for long-term objectives—the core components of grit. Simultaneously, the belief that change is self-directed fulfills the need for autonomy. As Self-Determination Theory posits, satisfaction of basic psychological needs promotes proactive growth and enduring persistence in activities (Deci and Ryan, 2013). Thus, a growth mindset, through its “challenge-as-opportunity” cognitive restructuring, drives individuals to internalize difficulties as fuel for “resilient growth,” fostering a transition from frustration-prone withdrawal to a “thriving-on-difficulty” mode of grit development.

### 4.3 The mediating role of effort belief

Supporting Hypothesis 3, effort belief was identified as a significant partial mediator. In the relationship between growth mindset and learning engagement. While a growth mindset constitutes a global implicit theory regarding the malleability of human attributes (Dweck, 2006), effort belief functions as a situated cognitive appraisal—a student’s perception of whether effort is effective and worthwhile within a specific learning context.

Our results suggest that holding a global belief that ability can be developed translates into concrete learning behaviors only when it manifests as a robust conviction that effort is the mechanism for that development. Blackwell et al. (2007) found that a growth mindset predicted changes in effort beliefs and subsequently mediated the relationship between effort beliefs and math achievement in middle schoolers. Jones et al. (2012) tested and extended Blackwell et al. (2007) motivational model with U.S. ninth-grade Algebra students, confirming the links among growth mindset, effort belief, and learning interest within a domain-specific framework. A meta-analysis by Tempelaar et al. (2015) further established effort belief as a “motivational hub” between mindset and academic outcomes. Similarly, Lou et al. (2017) longitudinal study demonstrated that a growth mindset significantly predicted effort belief in university students. Therefore, individuals with a growth mindset are more likely to hold strong beliefs that effort leads to improvement, prompting them to approach learning tasks with greater determination and, consequently, higher engagement.

### 4.4 The serial mediating pathway: grit and effort belief

Supporting Hypothesis 4, the present findings revealed a significant serial mediation effect in which growth mindset fosters learning engagement sequentially through grit and then effort belief. This result positions grit as a critical volitional bridge connecting the cognitive dispositions with subsequent behavioral outcomes.

This chain aligns with Duckworth et al. (2007) grit theory, in which highly gritty students demonstrate perseverance and a strong conviction in the utility of effort. Such students exhibit superior self-control, goal orientation, and resilience, viewing setbacks as learning opportunities.

From a positive psychology perspective, grit may function as a malleable character strength that potentially triggers a “cognition-behavior-affect” cascade. Students high in grit are more likely to perceive difficulties as challenges rather than threats, adopting what Dweck terms a “growth-oriented” mentality. Through sustained persistence, their implicit belief that “effort cultivates ability” may be reinforced, facilitating a shift from passive task compliance to active self-challenge. Conceptually, this interplay between belief and behavior could form an “efficacy-passion cycle,” representing a potential upward spiral from persistence to passion.

In summary, for primary school students, the data suggest a conceptual progression wherein a growth mindset relates to the cultivation of grit. This disposition appears to foster a stronger belief in the value of effort, which subsequently associates with deeper learning engagement. This serial pathway reveals the dynamic process by which cognitive dispositions translate into motivated behavior, providing educators with a theoretical lens for understanding the deeper drivers of student engagement.

### 4.5 The moderating role of teacher support

Supporting Hypothesis 5, the findings of this study revealed that perceived teacher support significantly moderates the pathway from growth mindset to grit among primary school students. This result extends previous literature, which has predominantly positioned teacher support as an antecedent or mediator in student engagement models (e.g., Sadoughi and Hejazi, 2023). From a developmental perspective, early adolescence is a critical period for identity formation and self-regulation (Eccles and Roeser, 2011). For primary students, teacher support functions not merely as a general resource but as a “scaffolding” mechanism that validates the risk-taking implicit in a growth mindset. As Haimovitz and Dweck (2017) noted, teachers’ incremental theory praise and supportive feedback create a classroom climate in which students feel safe to persist through challenges. In this study, when teacher support was high, students with a strong growth mindset were significantly more likely to develop grit; conversely, the positive predictive power of mindset diminished under low-support conditions. This suggests that a growth mindset alone is insufficient to foster perseverance in young learners unless it is embedded within a supportive interpersonal context.

The results also confirmed Hypothesis 6, indicating that perceived teacher support moderated the path from growth mindset to learning engagement. It aligns with the view that a growth mindset does not automatically translate into active learning behaviors for elementary students, but rather requires a supportive relational context to be activated (Dweck and Yeager, 2019; Haimovitz and Dweck, 2017). While a growth mindset provides the cognitive belief that ability can be developed (Dweck, 2006), young students often depend on external scaffolding to convert this belief into sustained engagement (Skinner and Edge, 2002; Furrer and Skinner, 2003). The results suggest that when teacher support is perceived as high—characterized by emotional encouragement, autonomy support, and clear structural guidance (Reeve, 2012; Ryan and Deci, 2020)—students are more likely to leverage their growth mindset to persist in tasks and participate actively. Conversely, in low-support classrooms, the potential link between mindset and engagement may diminish, as students might lack the psychological safety or clarity needed to take on learning challenges (Pianta et al., 2012; OECD, 2025). This moderating role highlights that, for primary school children, teacher support acts not only as a direct predictor of engagement but also as a critical condition that strengthens the ways in which personal motivational beliefs (like mindset) manifest in classroom behaviors (Theis and Fischer, 2022; Eccles and Roeser, 2011). These findings echo recent large-scale evidence indicating that supportive teacher practices amplify the mindset-outcome association, whereas the absence of such support can render mindset beliefs less effective in driving learning engagement (OECD, 2025; Cai, 2025).

## 5 Theoretical and practical implications

Theoretically, this study makes two interrelated contributions that extend current understanding of how growth mindset translates into learning engagement in the primary school context. First, by modeling multiple sequential mechanisms (grit → effort belief) simultaneously, this moves beyond treating these variables as independent mediators that grit functions as a psychological precondition through which effort beliefs contribute to sustained learning engagement. Second, examining multi-locus moderation reflects complex classroom reality. By testing moderation at multiple path-specific nodes rather than at the global level, this approach captures the nuanced reality that teacher support matters most precisely when growth mindset needs to be converted into persistent action.

Practically, the results argue against isolated mindset interventions. Effective programs should adopt a dual-channel approach, integrating student-level training targeting growth mindset, grit, and effort beliefs. Such training should follow a sequential design—first cultivating long-term perseverance through goal-setting and failure-coping exercises, then reinforcing the belief that effort drives improvement, rather than treating these three constructs as independent modules. Teacher-level professional development to foster autonomy-supportive, emotionally positive, and feedback-rich classroom climates. This development must explicitly train teachers to recognize and intervene at the critical transition point from a growth mindset to sustained effort, equipping them with strategies such as process-oriented praise, constructive responses to struggle, and scaffolding for students’ goal-pursuit.

## 6 Limitations and future directions

Several limitations must be acknowledged. First, the cross-sectional design precludes definitive causal conclusions. Although the proposed model was theoretically informed and evaluated using bootstrapping procedures, the temporal ordering of variables cannot be confirmed. Second, common-method variance may be a concern due to the reliance on self-reported measures. Third, the generalizability of findings may be limited to similar educational settings and age groups.

Future research should prioritize longitudinal or experimental designs to establish temporal and causal precedence. Using multi-informant or observational measures would mitigate common-method bias. Multilevel modeling could disentangle the effects of individual-perceived versus classroom-aggregated teacher support. Finally, investigating heterogeneity across grade levels, academic risk profiles, and demographic subgroups would provide a more nuanced understanding of these dynamics.

## 7 Conclusion

The present findings suggest that growth mindset is associated with learning engagement through a conditional, sequential mediation process involving grit and effort beliefs, with perceived teacher support serving as a contextual amplifier of these pathways. Consequently, a comprehensive account of student engagement necessitates integrating individual motivational dispositions with salient environmental supports. This integrative framework provides a conceptually grounded basis for designing educational interventions that are both psychologically informed and contextually sensitive, although future experimental or longitudinal study is required to establish causal efficacy.

## Data Availability

The raw data supporting the conclusions of this article will be made available by the authors, without undue reservation.
